# Epigenetic Modulation Directs Recovery Post LASIK and SMILE Surgery: An Experimental Study

**DOI:** 10.3390/life15020246

**Published:** 2025-02-06

**Authors:** Rohit Shetty, Pooja Khamar, Ramaraj Kannan, Puja Thacker, Nimisha Rajiv Kumar, Arkasubhra Ghosh, Vrushali Deshpande

**Affiliations:** 1 Cornea and Refractive Services, Narayana Nethralaya, Bangalore 560010, India; drrohitshetty@yahoo.com (R.S.); dr.poojakhamar@narayananethralaya.com (P.K.); 2 GROW Research Laboratory, Narayana Netralaya Foundation, Bangalore 560099, India; ramaraj@narayananethralaya.com (R.K.); pujathackerr@gmail.com (P.T.); nimisha@narayananethralaya.com (N.R.K.)

**Keywords:** epigenetics, SMILE, LASIK, hypermethylation, hypomethylation, refractive surgery

## Abstract

Purpose: refractive surgery, such as LASIK and SMILE, induces a wound healing response that leads to significant corneal stromal remodeling. We have shown that the protein profile in the stroma changes dramatically immediately post-surgery. However, the methylation status of the DNA post-refractive surgery remains unknown. Design/Participants: DNA methylation study. Refractive surgery (SMILE/LASIK) performed on donor eye globes. Method: we investigated the epigenetic changes post-surgery in relation to long term ECM remodeling in an experimental ex vivo study design. Donor globes (n = 19) were obtained from the eye bank. Three globes served as non-surgical controls while SMILE (-6DS) and LASIK surgery (-6DS) were performed on eight globes each and incubated for 3 days and 2 weeks (n = 4 per group per time point). Here, we compared the DNA methylation landscapes of LASIK and SMILE stroma using the Illumina Infinium Human Methylation 850 EPIC array (HM850). Results: significant changes in DNA methylation patterns were observed post-operatively in both LASIK and SMILE groups. Specific genes involved in the activation of actin cytoskeleton and inflammation (*smad3*, *prkca* and *ssh2*) showed hypomethylation in LASIK after 2 weeks and LASIK SMILE after 3 days, respectively, suggesting their active role in corneal repair. The genes (*gaa*, *gstm1*, *mgat1*, *galnt9* and *galnt5*) involved in sphingolipid metabolism and mucin biosynthesis showed hypomethylation in SMILE after 3 days. Conclusions: our results suggest that altered DNA methylation patterns may have relevance to the development of complications of haze post-refractive surgery. It also presents the opportunity to utilize drugs that regulate chromatin remodeling for optimal outcomes.

## 1. Introduction

Cornea is an avascular part of the eye which provides about 65 to 75% of the refractive power to the eye. It allows light to pass through it and focuses on the retina [1]. Refractive defects are mainly associated with the dysfunction of the cornea which, if not timely treated, could lead to severe discomfort in vision and, finally, vision loss. The altered disease phenotypes could be due to the interaction of several epigenetic mechanisms and genetic factors. Previously, it has been reported that heritability and epigenetics also play a role in the pathology of many ocular diseases along with mutation in the genes [2]. The role of epigenetic advances such as DNA methylation, chromatin remodeling, and non-coding RNAs has been recognized recently with respect to normal ocular development and various ocular diseases [3,4,5,6,7,8,9]. In cases where ocular diseases cannot be attributed to genetic mutations, epigenetics may play a significant role in understanding the disease pathogenesis [10,11,12,13]. Many ocular conditions are genetic and identical twin studies suggest variation in the heritability of ocular conditions: 27% for diabetic retinopathy [14] and 90% for myopia [15]. The epigenetic changes can be attributed to several environmental factors, and in-depth research is needed to understand the disease pathogenesis and design the epigenomic-targeted treatment modalities [13,16,17,18]. *Dnmts* has shown high levels of expression in the cornea and has been found to play an essential role in corneal wound healing [3]. The increased expression of *dnmt1* and *dnmt3b22* in a mouse model has been observed during corneal epithelial wound healing. Moreover, these subfamilies of dnmts may also control epithelial cell migration, differentiation, and proliferation [19]. The knockdown of *dnmt1* has been found to slow down the corneal epithelial wound healing in mice, signifying the importance of *dnmt1* methylation in wound healing processes.

Therefore, the objective of our study is to compare the DNA methylation landscapes of stroma 1 day and 2 weeks after LASIK and SMILE surgery. The main focus is to identify and characterize global DNA methylation changes in CpG islands and promoter regions in human corneas following refractive surgery and identify novel genes and pathways to understand the molecular mechanisms underlying post-surgical complications such as corneal haze.

## 2. Materials and Methods

### 2.1. Subject Collection

An experimental study was designed and approved by the Ethics Committee at Narayana Nethralaya Hospital. Informed consent from all the donors was obtained. All the surgical procedures were performed in accordance with the tenets of the Declaration of Helsinki.

### 2.2. Refractive Surgery on Donor Corneas

The refractive surgery (SMILE/LASIK) was performed 4–6 h after the procurement of cadaveric eyeballs (n = 19) stored in MK media in sterile conditions at 4 °C. All the surgeries were performed by a single experienced surgeon. The LASIK flap of 9.0 mm diameter and 110 µm thickness with a side cut angle of 70°, a canal width of 1.5 mm, and a hinge position at 90° was created with a femtosecond laser (Wave Light FS200 Femtosecond Laser and Wave Light EX500 Excimer Laser; Alcon Laboratories, Ft Worth, TX, USA). The device parameters were set to a 200 kHz repetition rate, a 1030 nm wavelength, and a 5 µm spot. The flap was created and manually lifted, and an excimer ablation of the targeted refraction OD-6DS (8.25 mm) was fired/provided using the Alcon/Wave Light EX500 excimer laser with the pupil centered using advanced eye tracking mode. The femtosecond laser settings were set to an 8 μm stromal bed cut spot separation, an 8 μm line separation, a 5 μm side cut bed separation, a 3 μm line separation, a 0.80 μJ bed cut pulse energy, and side cut pulse energy. The cornea was remoistened with a wet merocele sponge at the end of the procedure. The contralateral eye SMILE surgery was performed on a VisuMax femtosecond laser system (Carl Zeiss Meditec AG, Jena, Germany) with a 500 kHz repetition rate. For the creation of a lenticular side cut, spot spacing was 2 µm and 4.5 µm for the creation of a lenticular side cut with a preset laser cut, the energy was set to 170 nJ and the track distance to 3 mm. Cap thickness was kept as 110 µm, with a lenticule diameter of 6.0 mm and a cap diameter of 7.7 mm (optical zone). The refractive lenticule for correction-6DS (7.7 mm) was dissected and removed manually.

### 2.3. Sample Processing and Infinium 850 K Methylation Array

**Figure 1:**
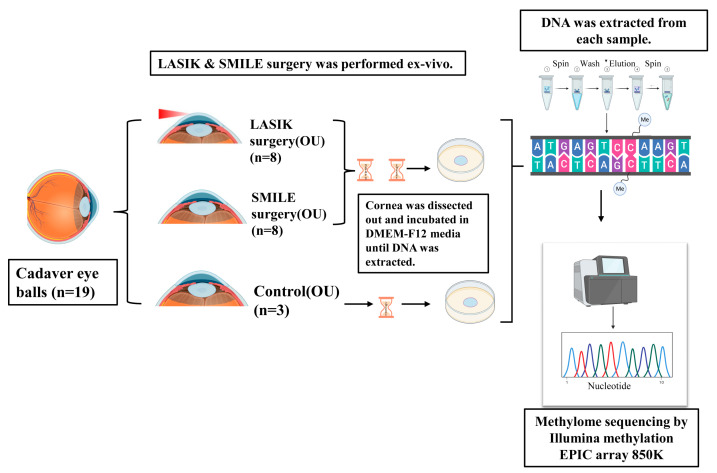
Schematic of the experimental design. LASIK and SMILE surgery was performed ex vivo in cadaver corneas (n = 19). Each cornea was dissected and incubated in DMEM-F12 media for 3 days and 2 weeks. A DNA sample was extracted and subjected to methylome sequencing by the Illumina Methylation Epic array 850 K.

After the surgery, the cornea was dissected out of the eyeballs and was cultured for 2 weeks and 3 days in DMEM-F12 media and kept in an incubator with 5% Co2 and 95% humidity. DNA was extracted using a Qiagen kit (Qiagen, Hilden, Germany) and integrity was examined by running a 1.3% agarose gel. Bisulfite modification of 5 µg genomic DNA was followed using the manufacturer’s protocol. DNA quality was accessed with the bioanalyzer Tapestation or agarose gel electrophoresis to ensure high quality, undegraded double stranded DNA. The control DNA was subjected to the same library preparation and sequencing workflow as the test DNA, with the exception of exclusion from the bisulfite treatment. After bisulfite conversion, gDNA from each sample was amplified and fragmented. The DNA was purified and put on to the bead chips. The DNA binds to DNA oligomers attached to beads. Further converted DNA was hybridized on an Infinium Human Methylation 850 Epic Array containing bead types for both Infinium I and II assays following the Illumina Infinium HD Methylation protocol. The chip was analyzed using an Illumina HiScan SQ fluorescent scanner (Illumina, San Diego, CA, USA), and the GenomeStudio Methylation module software v2.0.5 showed the intensities of the images. DNA methylation values were reported as beta values, usually represented as a continuous variable between 0 and 1. The differential methylation pattern was compared between control and test sample. Depending on the distance of the methylated loci from the nearest gene, the loci were categorized as CpG islands, shelves, or shores. The overall schematic of the study design is shown in Figure 1.

## 3. Statistical Analysis

### 3.1. Volcano Plot

The raw data were obtained after methylome sequencing as beta intensity values and average beta values for each group and different time points. The fold change values were calculated for the corresponding groups, followed by a log2 transformation. A student’s *t*-test was performed to find the significance value for the comparison between groups and the control. For a better representation of volcano plots, the *p*-values were transformed to −log10 values. The calculated log2 fold change value and −log10 *p*-value were plotted as a scatter plot in Graphpad Prism 8. The cut off for the differential expression was set to −2 and 2 (log2 scale) and the *p*-value to 1.3 (−log10 value).

### 3.2. Heirarchial Cluster Analysis and CpG Methylation Differences

The data were imported into Python v3.7 and the row-wise Z-score normalization was performed for a better visualization of the heatmap. The visualization library Seaborn v0.9.0. was used to generate the heatmap.

### 3.3. Pathway Analysis

The gene symbols were converted to gene IDs and used to search for pathways in the KEGG pathway analysis tool [20].

### 3.4. Gene Pathway Network

Functional enrichment analysis and gene and pathway interaction networks were constructed using Cytoscape. The colored nodes are the genes corresponding to multiple pathways. All the represented pathways are highly significant following a Benjamini–Hochberg correction. A pathway enrichment analysis and visualization were performed in Cytoscape, version 3.0.2 [21].

## 4. Results

### 4.1. DNA Methylation Signatures Post LASIK and SMILE Surgery

**Figure 2:**
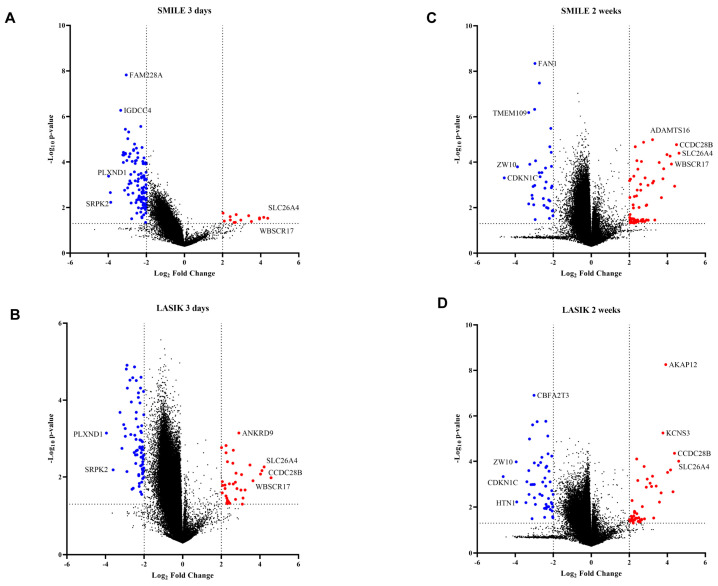
Volcano plot showing differences in methylation between the control and 3 days versus 2 weeks of time post LASIK and SMILE surgery (11). The graph was plotted with log2FC (fold change) (cut off < −2 to >2) and −log10 *p*-value < 0.05. The foldchange was calculated with respect to control samples (n = 3). (**A**) In SMILE 3 days (n = 4), the hypomethylated genes are shown as red circles and the hypermethylated genes are shown as blue circles. The top hits were *slc26a*4 and *plxnd1*. (**B**) In LASIK 3 days (n = 4), the hypomethylated genes are shown as red circles and the hypermetylated genes are shown as blue circles. The top hits were *slc26a*4 and *plxnd1*. (**C**) In LASIK 3 days (n = 4), the hypomethylated genes are shown as red circles and the hypermetylated genes are shown as blue circles. The top hits were *slc26a4* and *plxnd1*. (**C**) InSMILE 2 weeks (n = 4), the hypomethylated genes are shown as red circles and the hypermetylated genes are shown as blue circles. The top hits were *slc26a4* and *plxnd1*. (**D**) In LASIK 2 weeks (n = 4), the hypomethylated genes are shown as red circles and the hypermetylated genes are shown as blue circles. The top hits were *slc26a4* and cdkn1c.

To determine whether DNA methylation plays a role in wound healing post LASIK and SMILE surgery, we studied the CpG methylation profiles in the human cornea (n = 19 cornea). The volcano plot revealed all the significant hypermethylated and hypomethylated genes. The graph was plotted with log2FC (fold change) (cut off < −2 to >2) and a –log10 *p*-value < 0.05 (Figure 2). All the CpG sites with >±2 fold change values of β intensity were termed hypomethylated and hypermethylated with a *p*-value < 0.05 (Supplementary Table S1). Amongst the top hits, the *slc22a4* gene was hypermethylated in SMILE and LASIK surgeries after 3 days and 2 weeks (Figure 2A–D). The *wbscr17* gene was hypermethylated in SMILE after 3 days and 2 weeks and, in LASIK, after 3 days (Figure 2A–C). The *ccdc28b* gene was hypermethylated in SMILE for 2 weeks and in LASIK for 3 days (Figure 2B,C). Moreover, the *plxnd1* and *srpk2* genes were hypomethylated in SMILE and LASIK for 3 days (Figure 2A,B). The common genes that showed hypomethylation in SMILE and LASIK for 2 weeks were *zw10* and *cdkn1c* (Figure 2C,D).

### 4.2. Hypomethylated Genes Involved in Adaptive Immunity, Cell Migration, Transcription, and Protein Binding Across SMILE and LASIK

**Figure 3:**
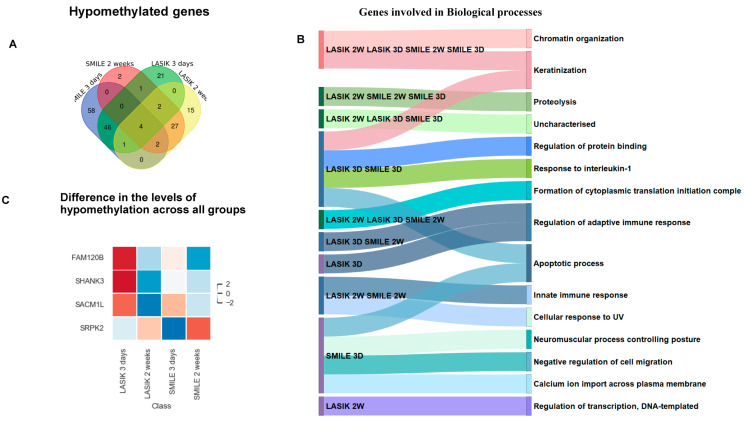
(**A**) Venn diagram showing common and unique hypomethylated genes across LASIK and SMILE at 3 days and 2 weeks. All the gene target IDs did annotate to the gene name. In SMILE 3 days, there were 58 unique target gene IDs. In LASIK 3 days, 21 gene IDs were uniquely expressed. LASIK 2 weeks showed the unique expression of 15 genes. There were 27 common gene IDs in LASIK and SMILE 2 weeks, whereas 46 common gene IDs were found in LASIK SMILE 3 days. Moreover, there were only two gene IDs in LASIK SMILE 2 weeks and LASIK 3 days. Only four gene IDs were common across all groups. (**B**) A Sankey diagram illustrating the shared and unique hypomethylated genes across different groups involved in different biological processes. (**C**) The heatmap shows variation in the expression levels of four common hypomethylated genes (*fam120b*, *shank3*, *sacm1l*, *srpk2*).

The Venn diagram represents the common and unique hypomethylated gene target IDs in SMILE and LASIK at 3 days and 2 weeks (Figure 3A). All the gene target IDs did annotate to gene name. In SMILE 3 days, 58 gene target IDs corresponding to 34 genes showed hypomethylation involved in apoptotic processes, neuromuscular processes, regulation of cell migration, and transcription. In LASIK 3 days, 21 gene target IDs corresponding to 10 genes were uniquely expressed and were involved in the regulation of the adaptive immune response; LASIK 2 weeks showed the hypomethylation of 15 gene target IDs corresponding to 10 genes involved in the regulation of transcription. There were 27 gene target IDs corresponding to 14 common genes found to be hypomethylated in LASIK and SMILE 2-week treatments and involved in the innate immune response and cellular response to UV, whereas 46 gene target IDs corresponding to 27 common genes were found to be hypomethylated in LASIK SMILE after 3 days and were involved in keratinization, regulation of protein binding, response to Interleukin-1, and apoptotic processes. Moreover, there were only two gene target IDs corresponding to one gene (*eif3a*) which showed hypomethylation in LASIK and SMILE after 2 weeks and in LASIK after 3 days and was found to be involved in proteolysis. Only four hypomethylated genes (*sp3*, *ankrd33b*, *kan*, and *banp*) were common across all groups and were found to be involved in chromatin organization and keratinization (Figure 3B). The heatmap shows the variation among all the four groups, representing the different levels of hypomethylation (Figure 3C).

### 4.3. Hypermethylated Genes Associated with Extracellular Matrix Organization and Inflammatory Response

**Figure 4:**
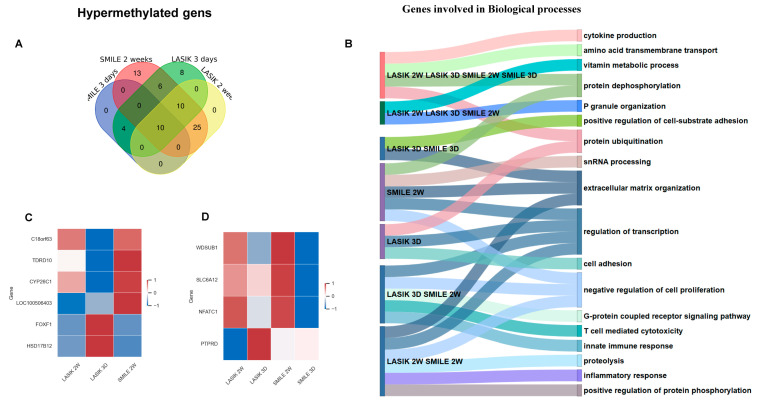
(**A**) Venn diagram showing common and unique hypermethylated genes across LASIK and SMILE at 3 days and 2 weeks. All the gene target IDs did annotate to gene name. In SMILE 3 days, there were no unique gene IDs. In LASIK 3 days, eight gene IDs were uniquely expressed. LASIK 2 weeks showed no uniquely expressed gene IDs. There were 25 common gene IDs in LASIK and SMILE 2 weeks, whereas 10 common gene IDs were found in LASIK SMILE 2 weeks and LASIK 3 days. There were 10 gene IDs common across all groups. (**B**) A Sankey diagram illustrating the shared and unique hypermethylated genes across different groups involved in different biological processes. (**C**) A heatmap shows the variation in the expression levels of four common hypomethylated genes across LASIK 3 days, 2 weeks and SMILE 2 weeks (*c18orf63*, *tdrd10*, *cyp26c1*, *loc100506403*, *foxf1*, *hsd17812*, *wdsub1*, *slc6a12*, *nfatc1*, *ptprd*). (**D**) A heatmap shows the variation in the expression levels of four common hypomethylated genes across LASIK and SMILE 3 days and 2 weeks (*wdsub1*, *slc6a12*, *nfatc1*, *ptprd*).

The Venn diagram represents the common and unique hypermethylated target gene IDs in SMILE and LASIK at 3 days and 2 weeks (Figure 4A). In SMILE 2 weeks, 13 targets and nine genes showed hypermethylation and were found to be mainly involved in extracellular matrix reorganization and cell proliferation. In LASIK 3 days, six genes were hypermethylated. However, there were no hypermethylated genes found in SMILE 3 days and LASIK 2 weeks. There were 14 gene target IDs corresponding to 10 hypermethylated common genes found in LASIK and SMILE 2 weeks, regulating extracellular matrix reorganization, cell proliferation, and the inflammatory response. Three genes, i.e., *foxf1*, *fendrr*, and *hsd17b*, were hypermethylated and shown to be involved in cell substrate adhesion and extracellular matrix organization. In LASIK SMILE 2 weeks and LASIK 3 days, 10 gene target IDs corresponding to four common genes (*c18orf63*, *tdrd10*, *cyp26c1*, and *loc100506403c18*) were hypermethylated and shown to be involved in metabolic processes and P granule organization, whereas, in SMILE 2 weeks and LASIK 3 days, six gene target IDs corresponding to five genes were hypermethylated. In total, 10 gene target IDs corresponding to four genes, i.e., *wdsub1*, *slca12*, *nfatc1*, and *ptprd*, were found to be hypermethylated across 3 days and 2 weeks in SMILE and LASIK and were found to be involved in cytokine production, protein ubiquitination, and vitamin metabolic processes (Figure 4B). However, the level of hypermethylation varied across the groups, as shown in the heat map (Figure 4C,D).

### 4.4. Significantly Methylated Genes Highlighting Vital Biological Pathways Post SMILE and LASIK Surgeries

A pathway–gene interaction network was constructed using Cytoscape 3.2.0 and analyzed based on the degree of interactions.

**Figure 5:**
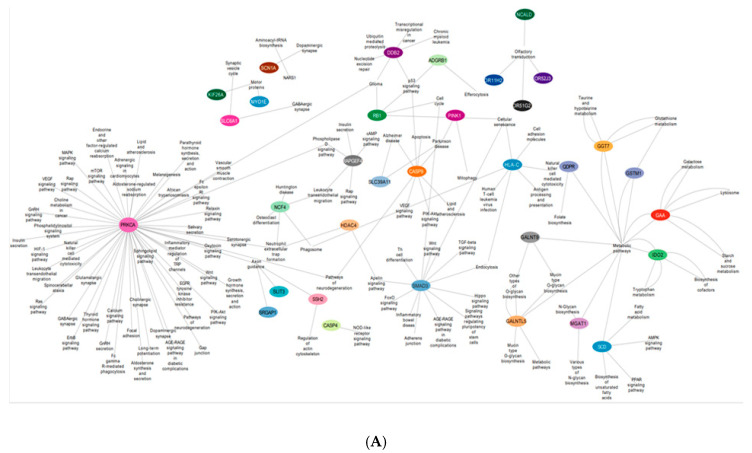

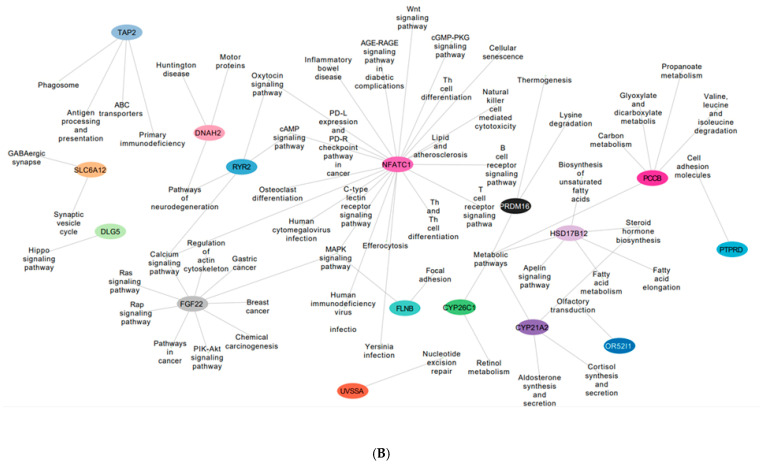
(**A**) An interactive gene–pathway network generated by Cytoscape comprising the genes (outlined in colored circles) that exhibited hypomethylation and the genes involved in multiple biological pathways. (**B**) An interactive gene–pathway network generated by Cytoscape comprising the genes (outlined in colored circles) that exhibited hypermethylation and the genes involved in multiple biological pathways.

Hypomethylated genes *ssh2*, *slit3*, and *srgap1* were found to be involved in the regulation of actin cytoskeleton and axon guidance, respectively, in SMILE and LASIK 3 days. The *Smad3* gene expressed in LASIK 3 weeks was involved in the Wnt signaling pathway, the TGFbeta signaling pathway, and the FOXO signaling pathway, and Th cell differentiation was hypomethylated. More importantly, the genes *galntl5*, *galntl9*, *mgat1*, and *gaa* showed hypomethylation in SMILE 3 days and were involved in metabolic pathways like mucitype O-glycan biosynthesis and galactose and glutathione metabolism. The hypomethylated HLA-C gene was involved in cell adhesion, cellular senescence, and NK cell mediated cytotoxicity. Hypomethylation of protein kinase C family gene *prkca* expressed in LASIK and SMILE 3 days showed to be involved in multiple pathways like calcium signaling, focal adhesion, mTOR, MAPK, and inflammatory mediator regulation of TRP channels (Figure 5A).

The hypermethylated genes *ryr2* and *dnah2* involved in the neurodegenerative pathway were found to be expressed in SMILE 3 days and LASIK 2 weeks. The gene *pccb* was found to be hypermethylated in LASIK SMILE 2 weeks and was involved in carbon and propanoate metabolism. In LASIK and SMILE 2 weeks, the gene *fgf22* was hypermethylated and involved in calcium signaling, the regulation of actin cytoskeleton, and the Ras signaling pathway. The gene *nfatc1* was shown to be involved in many pathways such as the Wnt signaling pathway, the cGMP PKG signaling pathway, Th cell differentiation, and calcium and MAPK signaling pathway across all groups (Figure 5B).

### 4.5. Discovery of DNA-Methylated CpG Islands Linked to the Process of Wound Healing over a Span of 3 Days and 2 Weeks in Both SMILE and LASIK Procedures

**Figure 6:**
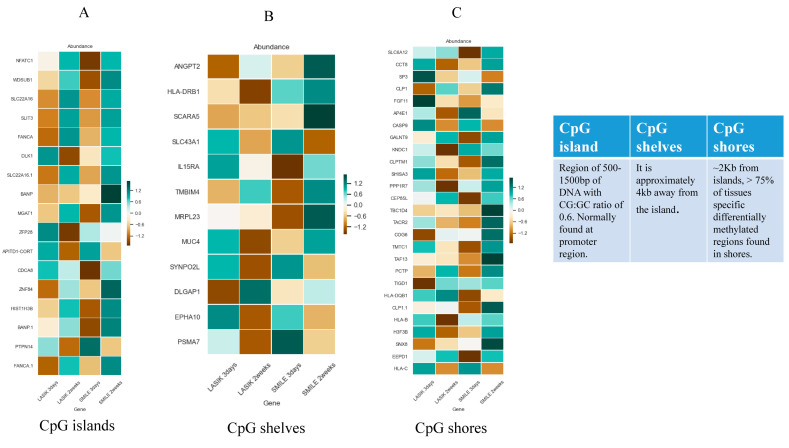
(**A**) Heat map of differentially methylated CpG islands in the study group compared with the control group. (**B**) Heat map of differentially methylated CpG shelves in the study group compared with the control group. (**C**) Heat map of differentially methylated CpG shores in the study group compared with the control group. * DNA green denotes CpGs with the highest methylation levels and brown denotes CpGs with the lowest methylation levels.

Using stringent filter criteria, we could observe that very few genes were differentially expressed across 3 days and 2 weeks in SMILE and LASIK. We relaxed the filter criteria and observed significant differences in gene expression in CpG islands, CpG shelves, and CpG shores for both LASIK and SMILE. CpG islands are the region with a CG:GC ratio of 0.6 and they are mostly present in the promoter region with 1500–2000 bp of DNA. CpG shores are the regions that flank 2 kb of the CpG island and CpG shelves, which are 4 kb away from the CpG island but flank 2 kb from CpG shores, which are also found to be methylated dynamically. Figure 6A–C below shows the gene expression profiling in the CpG island (A), shelves (B), and shores (C). Seventeen genes in the CpG island, 12 genes in the CpG shelves, and 27 genes in the CpG shores showed a differential expression in LASIK and SMILE. In the CpG island, *wdsub1*, *slc22a16*, *slit3*, *fanca*, *mgat1*, *znf84*, and *hist1h3b* genes were hypomethylated in LASIK SMILE 3 days and hypermethylated in LASIK SMILE 2 weeks, indicating the differences across time points. However, *atpitd1-cort* and *ptpn14* genes were hypermethylated in LASIK SMILE 3 days and hypomethylated in LASIK SMILE 2 weeks. The gene *nfatc1* was hypomethylated in SMILE 3 days and hypermethylated in LASIK and SMILE 2 weeks. The gene *dlk1* was hypomethylated in LASIK 2 weeks and hypermethylated in Lasik 3 days and SMILE 2 weeks. The gene *cdca8* was hypomethylated in SMILE 3 days, while it was hypermethylated/non-significant in LASIK SMILE 2 weeks and LASIK 3 days. There was an alteration in the expression of *zfp26* in LASIK 3 days and LASIK 2 weeks, but it was not significant in SMILE. In the CpG shelves, *hla-drb1* showed a difference in the methylation status in LASIK and SMILE. The gene *scara5* was hypermethylated in SMILE 2 weeks only and was hypomethylated in other groups. The gene *il15ra* was hypomethylated in the SMILE 3 days group only and was hypermethylated in other groups. The gene *dlgap1* showed a difference in methylation status in LASIK 3 days and 2 weeks, but there was no significant change in the SMILE groups. The gene *mrpl23* was hypomethylated in SMILE 3 days and hypermethylated in SMILE 2 weeks, while there was no difference observed in the LASIK groups. Major alterations in the methylation status in the genes were observed in CpG shores. The genes *slca12*, *galnt9*, *cep65l*, *hla-dqb1*, and *eepd1* were hypomethylated in SMILE 3 days and hypermethylated in other groups. The genes *hla-b*, *pp1r7*, and *kndc1* were hypomethylated in LASIK 2 weeks but hypermethylated in other groups. The genes *tigd1* and *cog6* were hypomethylated in LASIK 3 days but hypermethylated in other groups. Four genes, i.e., *taf13*, *tacr2*, *tbc1d4*, and *cld1*, were hypermethylated in SMILE 2 weeks but did not show any difference in other groups.

## 5. Discussion

Our understanding of DNA methylation with respect to ocular conditions is rapidly progressing. The cornea, with its high expression of DNA methyltransferases [3] (*dnmts*), is involved in corneal epithelial wound healing, organizing processes such as the regulation of epithelial cell migration, differentiation, and proliferation [19]. Beyond corneal epithelial wound healing, *dnmts* exhibit significance in lens development, as evidenced by the demethylation of the γ-crystallin promoter gene in a rat model [4]. Recent investigations in zebrafish [22] and mouse models [5] have further substantiated the critical role of *dnmt1* in lens development. Inactivation of *dnmt1* during embryonic development leads to apoptosis, diminished proliferation of lens cells, and anomalies in lens development, emphasizing the pivotal role of DNA methylation in ocular biology [5,22].

Subsequent investigations [23] have demonstrated that demethylation has a positive impact on both mRNA and protein levels of γ-crystallin [23]. More recent research has unveiled alterations in DNA methylation associated with age-related cataracts [24] (ARC). Notably, hypermethylation of 8-oxoguanine DNA glycosylase 1 (*ogg1*) has emerged as a contributing factor to cataract development. In 2017, a group of researchers successfully demethylated *ogg1*, leading to an upregulation in *ogg1* expression and causing the protection of lens cells from UVB light to induce apoptosis [25]. This same group identified five additional genes, mainly DNA methyl transferases and histone deacetylases, with heightened expression in ARC cells, suggested to play a role in catalyzing epigenetic changes in cataracts [25]. In the context of glaucoma, where fibrosis of the trabecular meshwork (TM) diminishes the aqueous humor outflow, resulting in elevated intraocular pressure [26] (IOP), recent studies propose that epigenetic modifications regulate the delicate balance between profibrotic and anti-fibrotic factors. Notably, the RAS protein activator like 1 (RASAL1) has been implicated as a key player in this epigenetic modulation [26].

Fuchs endothelial corneal dystrophy (FECD) has shown DNA methylation changes involved in fluid transport and metabolism defects [27,28]. However, the activation of cytoskeletal-organization-associated genes has been shown to protect the corneal stroma from the accumulation of excess fluid [13].

The present study highlights epigenetic modifications, particularly alterations in DNA methylation, within the stroma of individuals undergoing LASIK and SMILE surgeries. Employing an extensive, genome-scale DNA methylation array, visible differences in the DNA methylation profiles were observed between LASIK and SMILE procedures in comparison to patients with normal vision. Notably, a substantial number of genes exhibited hypomethylation patterns in both SMILE and LASIK patients across the 3-day and 2-week postoperative periods. A gene ontology analysis further elucidated DNA methylation alterations in genes associated with cytoskeletal organization, metabolic processes, inflammatory pathways, cell cycle regulation, and cellular processes. These findings propose that changes in DNA methylation may play a role in shaping distinct wound healing responses by influencing the metabolic, cytoskeletal organization and inflammatory reactions within the stroma following LASIK and SMILE surgeries.

Promoter DNA hypermethylation has been correlated with the transcriptional silencing of genes [29]. Conversely, atypical gene-specific demethylation and widespread hypomethylation, affecting duplicate sequences across the genome, have the potential to induce the overexpression of genes and activate transposable elements, thereby contributing to disease pathogenesis [30]. Notably, our observations revealed significant promoter DNA hypermethylation in LASIK at the 3-day mark for the *dnah2* gene and in SMILE at the 2-week interval for the *ryr2* gene, both implicated in pathways associated with neurodegeneration.

Of particular interest, the deletion of *Ryr2* was found to inhibit unfolded protein response/endoplasmic reticulum (ER) stress, activate the CCAAT-enhancer-binding protein homologous protein, and stimulate the cyclic adenosine monophosphate response element-binding protein. This cascade of events led to an initial cone protection, shedding light on the intricate molecular mechanisms underlying the impact of DNA methylation on neurodegenerative pathways [31].

Significant modifications in the hypomethylated status of genes were observed in the small incision lenticule extraction (SMILE) procedure at an early time point compared to laser-assisted in situ keratomileusis (LASIK). Predominant alterations occurred in gene families associated with mucin type O glycan biosynthesis, metabolic pathways, and sphingolipid metabolism, with the notable involvement *of gaa*, *gstm1*, *mgat1*, *galnt9*, and *galnt5.* Mucins and glycoproteins synthesized in the corneal epithelial cells play a crucial role in maintaining normal vision. Specifically, O-glycans on epithelial cell mucins exert control over antigenicity and the conformation of mucin molecules. The contribution of O-glycans extends to microbial attachment, thereby preventing their invasion into underlying cells, including viruses [32] and bacteria such as *Staphylococcus aureus*, a main contributor to ocular infections [33]. These observations suggest a potential reduction in the risk of infection and postoperative dry eye following SMILE surgery.

During the onset of corneal wound healing, a surge in metabolic activity and the restructuring of cell architecture occurs, mirroring observations in our data [34]. The complex process of corneal wound healing involves the migration of inflammatory cells and keratocytes, drawn to the wound site through chemotaxis, culminating in the remodeling of the extracellular matrix [35]. An overabundance of proliferation and transdifferentiation in corneal stromal fibroblasts can lead to haze and fibrosis in the cornea. The transforming growth factor beta (TGFβ) plays a crucial role in corneal repair and fibrosis, transmitting its signals through the Smad pathway. A study utilizing a corneal repair model demonstrated that in Smad3 knockouts, wound-induced fibronectin accumulation mirrors that of wild-type corneas, while the transformation into myofibroblasts is diminished in Smad3 knockout model. Primary cultures of corneal stromal cells in the presence of TGFβ have revealed that Smad3-deficient cells exhibit decreased efficiency of myofibroblast transformation without a concurrent decrease in fibronectin expression. Consequently, Smad3 deficiency leads to a selective decrease in fibrotic markers during corneal repair [36].

In our observations, the hypomethylation of the *smad3* gene in LASIK after 2 weeks suggests the activation of the TGFβ pathway, potentially increasing pro-fibrotic molecules. This activation may contribute to the development of corneal haze following LASIK. Following the LASIK and SMILE procedures, hypomethylation of *prkca* and *ssh2* genes activated both actin cytoskeleton and inflammatory pathways within 3 days. This finding aligns seamlessly with previous proteomic analyses, underscoring the early biological responses post-LASIK and SMILE, wherein the regulation of actin cytoskeleton and inflammatory responses was evident [37]. Alterations in the DNA methylation status of specific genes are implicated in the intricate modulation of the response to injury [38,39,40]. The significant hypermethylation of the *nfatc1* gene across both time points post SMILE and LASIK surgery suggests it is activation and is reported to be involved in many wound healing processes such as inflammation and angiogenesis [41]. The CpG island (CGI) region in the DNA methylation of gene promoters play a major role in gene regulation [42]. The genes involved in DNA binding, DNA damage and repair, and transcription regulation showed higher activity at the early time point (3 days) in LASIK and SMILE, a phenomenon which may be a response to injury.

This study has certain limitations, including the small number of corneas undergoing surgeries and of the control samples analyzed. Nonetheless, this investigation presents a pioneering genome-scale examination of DNA methylation in the context of SMILE and LASIK procedures. Our results reveal consistent DNA methylation alterations in the corneal stroma following refractive surgery, with distinct patterns observed post-LASIK compared to SMILE. These identified changes have the potential to influence the corneal wound healing process.

## 6. Conclusions

Notably, we pinpoint significant DNA methylation modifications in gene families associated with cytoskeletal organization, cellular metabolism, and inflammatory pathways connected to the cascade of wound healing. In light of the increasing development of drugs targeting DNA methylation over recent decades, primarily with respect to cancer therapies [43], our findings propose that altered DNA methylation could emerge as a novel and promising therapeutic target. Such interventions may hold the potential to enhance visual outcomes following refractive surgery.

## Data Availability

The raw data supporting the conclusions of this article will be made available by the authors on request.

## Supplementary Materials

The following supporting information can be downloaded at: https://www.mdpi.com/article/10.3390/life15020246/s1, Table S1: List of significant hypomethylated and hypermethylated genes post LASIK and SMILE surgery.
